# Increased Active OMI/HTRA2 Serine Protease Displays a Positive Correlation with Cholinergic Alterations in the Alzheimer’s Disease Brain

**DOI:** 10.1007/s12035-018-1383-3

**Published:** 2018-10-25

**Authors:** Taher Darreh-Shori, Sareh Rezaeianyazdi, Erica Lana, Sumonto Mitra, Anna Gellerbring, Azadeh Karami, Nenad Bogdanovic, Christina Unger Lithner, Bengt Winblad, Homira Behbahani

**Affiliations:** 1 Division of Clinical Geriatrics, Center for Alzheimer Research, NVS Department, Karolinska Institutet, Neo7th floor, 141 83 Huddinge, Sweden; 2 Division of Neurogeriatrics, Center for Alzheimer Research, NVS Department, Karolinska Institutet, 141 83 Huddinge, Sweden; 3 Neurogeriatric Clinic, Karolinska University Hospital, Center for Alzheimer Research, NVS Department, Karolinska Institutet, Huddinge, Stockholm Sweden

**Keywords:** Alzheimer’s disease, OMI/HTRA2, Cholinergic markers, NGF, APP, α7 nicotinic acetylcholine receptor

## Abstract

OMI/HTRA2 (high-temperature requirement serine protease A2) is a mitochondrial serine protease involved in several cellular processes, including autophagy, chaperone activity, and apoptosis. Few studies on the role of OMI/HTRA2 in Alzheimer’s disease (AD) are available, but none on its relationship with the cholinergic system and neurotrophic factors as well as other AD-related proteins. In this study, immunohistochemical analyses revealed that AD patients had a higher cytosolic distribution of OMI/HTRA2 protein compared to controls. Quantitative analyses on brain extracts indicated a significant increase in the active form of OMI/HTRA2 in the AD brain. Activated OMI/HTRA2 protein positively correlated with stress-associated read-through acetylcholinesterase activity. In addition, α7 nicotinic acetylcholine receptor gene expression, a receptor also known to be localized on the outer membrane of mitochondria, showed a strong correlation with OMI/HTRA2 gene expression in three different brain regions. Interestingly, the activated OMI/HTRA2 levels also correlated with the activity of the acetylcholine-biosynthesizing enzyme, choline acetyltransferase (ChAT); with levels of the neurotrophic factors, NGF and BDNF; with levels of the soluble fragments of amyloid precursor protein (APP); and with gene expression of the microtubule-associated protein tau in the examined brain regions. Overall, the results demonstrate increased levels of the mitochondrial serine protease OMI/HTRA2, and a coherent pattern of association between the activated form of OMI/HTRA2 and several key proteins involved in AD pathology. In this paper, we propose a new hypothetical model to highlight the importance and needs of further investigation on the role of OMI/HTRA2 in the mitochondrial function and AD.

**Electronic supplementary material:**

The online version of this article (10.1007/s12035-018-1383-3) contains supplementary material, which is available to authorized users.

## Introduction

OMI/HTRA2 is a mitochondrial serine protease and member of the high-temperature requirement serine protease A (HtrA)-chaperone family [1]. It is nuclear encoded and synthesized in the nucleus as a precursor protein containing a mitochondrial target sequence. When imported into the mitochondria, it is processed into the mature form and resides in the mitochondrial inter-membrane space (IMS). Upon apoptotic stimuli, OMI/HTRA2 is released from the mitochondria into the cytosol where it interacts with the cytosolic inhibitor of apoptosis proteins (IAPs) and prevents their caspase-inhibition function [2, 3].

OMI/HTRA2 is important for several cellular processes including mitochondrial function, autophagy [4], chaperone activity [5], and apoptosis [6]. It promotes cell survival by the maintenance of mitochondrial homeostasis [7–9], while under stressful condition it may switch from a protector into pro-apoptotic factor [10–13].

OMI/HTRA2 has been implicated in the pathogenesis of several neurodegenerative disorders [9, 14, 15]. However, it is unclear if it is the protease activity itself of OMI/HTRA2 or its pro-apoptotic function via interaction with members of the IAP family that is involved in neuronal cell death in neurodegenerative disorders. The mechanisms of cell death involved in these disorders have not yet been fully elucidated. The role of OMI/HTRA2 in neuronal death induced by status epilepticus (SE) has previously been studied in the immature rat brain [16]. Immunohistochemical examinations of the brain of this animal model together with western blot analyses of cytosolic and mitochondrial fractions have demonstrated cytosolic accumulation of OMI/HTRA2, induced X-linked IAP degradation, and enhanced caspase-3 activity in the post-SE hippocampal CA1-subfield [16], supporting a role of a pro-apoptotic function of OMI/HTRA2 in neuronal death. In addition, several recent studies have demonstrated that overexpression of OMI/HTRA2 markedly increases apoptosis [17, 18]. However, other studies on human primary neurons as well as on retinoic acid-differentiated cells, where the cells were exposed to certain stressors, have shown low numbers of caspase-3-positive cells despite an observed release of OMI/HTRA2 into the cytosol [16, 19]. This may indicate that the release of OMI/HTRA2 does not always correlate with or is related to apoptotic cascades in the cells and may have a pleiotrophic function depending on the stimuli.

It needs to be further elucidated whether the pro-apoptotic function or the protease activity of OMI/HTRA2 is involved in the pathogenesis of neurodegenerative disorders. The importance of OMI/HTRA2 becomes evident from an OMI/HTRA2 gene deletion study, showing a dramatic loss of striatal neurons, i.e., a neurodegenerative feature of Parkinson’s disease (PD) [9]. Another example comes from a motor neuron-deficient (*mnd2*) mice model exhibiting a similar phenotype and underlying pathology, which is found to be related to a Ser276Cys mutation in the encoding region of the *Omi/HtrA2* gene [7]. As this mutation strongly impairs the proteolytic activity of OMI/HTRA2, the loss of this activity most likely causes the pronounced phenotype of both *OMI/HTRA2*-deficient and *mnd2* mice. Additional links come from gene association studies. For instance, in a German PD patient population, the mutations A141S and G399S in the *OMI/HTRA2* gene have been identified and functional studies indicated that both polymorphisms impair the protease activity of OMI/HTRA2 and induce mitochondrial dysfunction and altered morphology [15]. It is noteworthy that a novel OMI/HTRA2 inhibitor has been found to exert neuroprotection by altering caspase-8 and caspase-3 cleavages in rats following ischemia/reperfusion brain injury [20].

OMI/HTRA2 protease function might also have a role in neuronal damage in Alzheimer’s disease (AD). An association between AD and the A141S genotype of OMI/HTRA2, which causes defective OMI/HTRA2 protease activity, has been previously reported by us in a Swedish AD case-control study [21]. We have also reported reduced level of the processed form (putatively active form) of OMI/HTRA2 in the frontal cortex of patients with AD compared to controls, as assessed in two separate study cohorts [21]. Intriguingly, despite the overall reduction in the OMI/HTRA2 protein levels, functional analyses indicated a significant increase of OMI/HTRA2 protease activity in the frontal cortex of the AD brain compared to control [21].

One of the main features of AD is an early deficit in the cholinergic signaling [22]. This feature is also shared by other AD-like dementia disorders, such as Lewy body dementia and Down’s syndrome [23, 24]. It is noteworthy that the maintenance and survival of cholinergic nuclei in the basal forebrain require nerve growth factor (NGF), the level of which is reduced in the brain of patients with AD [25].

The enzyme, choline acetyltransferase (ChAT), is responsible for the biosynthesis of the cholinergic neurotransmitter acetylcholine (ACh). In contrast, the cholinesterases, acetylcholinesterase (AChE) and butyrylcholinesterase (BuChE), degrade ACh within the synaptic cleft and in extracellular fluids. ChAT defines cholinergic neurons and is thereby the most specific cholinergic marker. Nonetheless, we have shown in a recent report that ChAT is also present in the human extracellular fluids, such as cerebrospinal fluid (CSF) and plasma, and is expressed and released by astrocytes, lymphocytes, and human embryonic stem cells [26].

Recent studies indicate that apart from its function as neurotransmitter, ACh also possesses an anti-inflammatory effect, for which the name “cholinergic anti-inflammatory pathway (CAP)” has been coined [27]. The anti-inflammatory action of ACh seems to be mediated through α7 nicotinic acetylcholine receptors (α7nAChR) [28]. Thus, acetylcholine should not be merely considered an important neurotransmitter for the cholinergic neuronal circuitries with a prominent role in various cognitive domains, but also may be a potential key player in the regulation of astroglial function under both normal and pathologic inflammatory conditions, such as in the AD brain. Indeed, depending on the stage of AD, inflammatory molecules and immune-competent cells, such as activated microglia and astrocytes, neutrophils, and other cell types, are abundant and/or transiently appear in lesion sites in the AD brain [29]. Thus, chronic inflammation seems to be another feature of the AD brain [30].

It is noteworthy that the biosynthesis of ACh demands stoichiometric amounts of the high-energy molecule acetyl-coenzyme A (A-CoA), which is believed to be mainly produced by glycolysis in mitochondria. Part of the produced amount of A-CoA is then entered and consumed within the citric acid cycle (also known as tricarboxylic acid cycle or Krebs cycle) for molecular energy conservation as, e.g., ATP. Thus, ACh biosynthesis is directly linked to the bioenergetic function of mitochondria. In addition, inflammatory processes may mediate the release of certain pro-apoptotic mediators from mitochondria, for example, the release of cytochrome c by nuclear factor kappa B-mediated inflammation [31]. Similarly, treatment of differentiated neuroblastoma cells and human primary neurons with interleukine-1β increases the release of OMI/HTRA2 from mitochondria, accompanied by increased OMI/HTRA2 protease activity. However, these changes do not necessarily lead to apoptosis [19].

Overall, very few studies on the level of OMI/HTRA2 expression in different regions of the AD brain are available and, to the best of our knowledge, none on its relationship with cholinergic markers and/or neurotrophic factors such as NGF. Considering the early cholinergic degeneration in AD, and the implication of OMI/HTRA2 in the pathogenesis of neurodegenerative disorders, mitochondrial function, and apoptotic events, we investigated the changes in cellular localization, protein levels, and gene expression of OMI/HTRA2 in different brain regions from AD and controls. Moreover, we were particularly interested to study whether changes in OMI/HTRA2 expression levels correlated with the levels of different cholinergic markers, AD-related genes such as amyloid precursor protein (*APP*) and microtubule-associated protein tau (*MAPT*), and neurotrophic factors such as NGF and brain-derived neurotrophic factor (*BDNF*).

## Materials and Methods

### Human Postmortem Brain Tissue

The human postmortem brain samples used for immunohistochemistry were provided by the Swedish Brain Bank. All studies involving human subjects had been approved by the Regional Ethical Review Board in Stockholm or the Research Ethics Committee of the South Huddinge University Hospital. Brain tissues from the frontal cortex (FC) of AD cases (*n =* 3, age 66.3 ± 5.1, postmortem delay 23.7 ± 16 h) and non-demented controls (*n =* 3, age 75 ± 6.1, postmortem delay 26.7 ± 9 h) were used.

The human postmortem brain samples used for gene expression and protein expression/activity analyses were provided by the Netherlands Brain Bank (NBB, Amsterdam, The Netherlands). The postmortem delay (PMD) was between 3 and 9 h. The NBB abides by the Dutch law for obtaining and using human tissues for scientific research. Brain tissues from the medial frontal gyrus (MFG), superior parietal gyrus (SPG), and superior temporal gyrus (STG) of AD cases (*n* = 6, age 80.5 ± 8.5, postmortem delay 5 ± 1 h, Braak stage V–VI) were used and compared to those of age-matched non-demented controls (*n* = 6, age 80.2 ± 7.9, postmortem delay 7.7 ± 1.6 h). Tissues were stored at − 80 °C until use. Additional information is presented in Table 1**.**

**Table 1:** Clinical information of controls and AD

	Brain bank	Sample number	Brain region	Age (years)	Sex	Disease duration (years)	Braak stage	PMD (hours)	Other information
Ctrl	NBB	1995-054	MFG, SPG, STG	72	f	–	I	9.2	
Ctrl	NBB	1996-085	MFG, SPG, STG	84	m	–	I	9.0	
Ctrl	NBB	2008-027	MFG, SPG, STG	80	f	–	I	7.0	
Ctrl	NBB	2008-032	MFG, SPG, STG	71	m	–	II	8.9	
Ctrl	NBB	2008-054	MFG, SPG, STG	92	f	–	I	7.0	
Ctrl	NBB	2009-005	MFG, SPG, STG	82	m	–	I	5.2	
AD	NBB	2004-068	MFG, SPG, STG	72	f	5	VI	6.5	
AD	NBB	2005-040	MFG, SPG, STG	69	m	9	VI	5.0	
AD	NBB	2006-059	MFG, SPG, STG	91	f	11	VI	3.75	
AD	NBB	2007-052	MFG, SPG, STG	82	m	8	V	4.25	
AD	NBB	2007-073	MFG, SPG, STG	87	m	12	V	6.1	
AD	NBB	2008-004	MFG, SPG, STG	82	f	6	VI	4.3	
Ctrl	SBB	150	FC	72	m	–	n.d.	18	
Ctrl	SBB	116	FC	71	m	–	n.d.	26	
Ctrl	SBB	147	FC	82	f	–	n.d.	36	
AD	SBB	202	FC	65	f	16	n.d.	23	
AD	SBB	212	FC	62	m	9	VI	40	APP-mutation
AD	SBB	215	FC	72	f	n.d.	VI	8	

*Ctrl* non-demented control, *AD* Alzheimer’s disease, *NBB* Netherlands Brain Bank, *SBB* Swedish Brain Bank, *MFG* medial frontal gyrus, *SPG* superior parietal gyrus, *STG* superior temporalis gyrus, *FC* frontal cortex, *PMD* postmortem delay, *n.d.* not determined

### Immunohistochemistry on Brain Sections

For immunohistochemistry, frontal cortex tissue blocks were fixed in buffered 4% formaldehyde and embedded in paraffin. Cryostat 7-μm-thick sections were mounted onto alum-gelatin-coated slides. The mounted sections were baked at 58 °C for 30 min, de-paraffinized, and hydrated. The sections were pre-treated with 3% H_2_O_2_ in tris-buffered saline (TBS, pH 7.6) for 10 min, followed by blocking of non-specific sites with DAKO-protein block for 30 min. After blocking, the sections were incubated with primary antibody against OMI/HTRA2 (R&D Systems, diluted 1:250) at 4 °C overnight. After this, sections were incubated with biotinylated horse anti-rabbit antibody (Vector Laboratories, Burlingame, UK), diluted to 5.0 μg/mL in TBS, for 30 min, followed by incubation in ABC-Elite HRP (Vector Laboratories, Burlingame, UK) for 1 h. Reactions were visualized by developing the sections in DAB (DAKO Cytomation, Denmark). All sections were treated simultaneously under the same conditions. The protocol for antibodies was repeated to assure reproducibility of results. For control staining, the primary antibody was omitted. Sections were counterstained with hematoxylin.

### RNA Extraction

Total RNA was extracted from ~ 80 mg of frozen brain tissue, with the RNeasy Plus Universal Mini Kit (QIAGEN), according to the manufacturer’s recommendations. After extraction, 1 μg of total RNA was treated with 1 U of DNAse I (Invitrogen) and then reverse transcribed into cDNA with the High Capacity Reverse Transcription Kit (Applied Biosystems), according to the manufacturer’s protocol.

### Gene Expression

To measure gene expression, 1/20th of the cDNA (obtained in the previous step) was used as a template for real-time PCR in a StepOne Plus thermocycler (Applied Biosystems) using the Gene Expression Master Mix (Applied Biosystems) and the following Taqman gene expression assays: Hs01553639_g1 (*HTRA2*), Hs00163746_m1 (*BCHE*), Hs00241307_m1 (*ACHE*), Hs02718934_s1 (*BDNF*), Hs00171458_m1 (*NGF*), Hs00213484_m1 (*MAPT*), Hs01063373_m1 (*CHRNA7*), and Hs01552291_m1 (*APP*). *GUSB* and *RPL13* (Taqman assays Hs99999908_m1 and Hs00744303_s1 respectively) were used as reference genes for normalization. All samples were run in triplicates. Relative gene expression values were calculated by the StepOne Software 2.0 based on a relative standard curve (serial dilutions of a pooled cDNA sample were used as standard), and were then normalized by dividing them by the geometric mean of the reference gene values.

### Brain Homogenates of AD and Control

Three consecutive extracts of the brain tissue homogenates were prepared. Approximately, 100 mg wet brain tissue was homogenized for 2–5 min, first in 1.5 mL (1:15 ratio) of Na/K phosphate buffer (50 mM, pH 7.4, containing 2 mM EDTA) to extract soluble proteins. After centrifugation for 30 min at 15,000 rpm at 4 °C, the supernatants were carefully collected and aliquoted in new Eppendorf tubes and kept frozen at − 80 °C. These extracts were used to quantify *soluble protein* fractions in the brain by sandwich enzyme-linked immunosorbent assays (ELISA).

The pellets were then re-homogenized in 1.5 mL (1:15 original ratio) of the second Na/K phosphate buffer, containing 500 mM NaCl, to extract salt-soluble *ionic proteins* from the brain tissues. The supernatants were collected and handled as above. The pellets were re-homogenated once more in 1.5 mL of a third Na/K-phosphate buffer, containing additional 0.6% Triton X-100, to extract the *membrane-bound proteins*. The supernatants were collected and handled as above. The total amount of proteins in each extract was determined using a protein assay kit (Pierce BCA; VWR International AB, Stockholm, Sweden).

### Measurement of Active OMI/HTRA2 Protein Level by In-House-Developed ELISA

A sandwich ELISA assay was set up and used to quantify the protein level of the activated form of OMI/HTRA2 in the brain extract from three different brain regions of patients and controls. Briefly, a 384-well plate (Nunc MaxiSorp, Cat. #464718) was coated with 50 μL/well of 0.25 μg/mL of capturing antibody (rabbit polyclonal anti-OMI/HTRA2 antibody, cat#AF1458; R&D Systems, UK) in coating buffer (100 mM carbonate buffer, pH 9.8, and containing 0.01% thimerosal as preservative), and incubated overnight at 4 °C under gentle orbital shaking. On the next day, the plate was washed once with TBS buffer and blocked with 75 μL/well of 4% BSA in coating buffer (carbonate buffer, pH 9.8) at room temperature (RT) for 1 h. The plate was then washed 3 × 5 min with TBS-T buffer (TBS, containing 0.1% Tween 20). The standards and the brain extracts were added to the wells (50 μL/well, all in triplicates) and incubated at RT for 2 h under gentle orbital shaking. The standard protein was recombinant human OMI/HTRA2 protein (Cat. #1458-HT; R&D Systems). Serial twofold dilutions were prepared ranging from S_1_ to S_16_, starting from S_1_ = 1 μg/mL of the recombinant protein. All the brain samples and standards were diluted in TBS-T-BSA buffer pH 7.4 (10 mM Tris HCl; 154 mM NaCl; 1 mM EDTA; 0.05% Triton-X100; 0.1% BSA).

The plate was then washed 3 × 5 min with TBS-T and incubated at RT for 3 h with 50 μL/well of a 1 μg/mL solution of a mouse monoclonal anti-OMI/HTRA2 antibody (active form; cat#MAB1458, R&D Systems, UK) in TBS-T buffer containing 4% BSA and 0.01% NaN_3_. After washing as before, the plate was incubated for 2 h at RT with 50 μL/well of a solution of alkaline phosphatase (AP)-conjugated bovine anti-mouse secondary antibody (sc-2377, Santa Cruz Biotechnology, 1:5000 in TBS-T). The plate was then washed 4 × 5 min with TBS-T and 1 × 5 min with diethanolamine buffer (DEA buffer pH 9.8 consisting of 225 mM diethanolamine, 1 M MgCl, and 0.01% NaN_3_). Finally, the plate was incubated with the fluorogenic substrate 4-methylumbelliferyl phosphate solution (80 μM in DEA buffer) and read kinetically at 1-min intervals using excitation 385 nm and emission 448 nm. The reaction was monitored with an Infinite M1000 Tecan microplate reader.

### Measurement of ChAT Activity and Protein Concentration

The activity and protein concentration of ChAT were assessed by an integrated assay in each brain extract essentially according to the procedure described previously [30]. Briefly, 10 μL of the brain extracts was transferred (in triplicates) to the wells of a 384-well ELISA plate (Nunc Maxisorp) that was precoated with mouse anti-ChAT monoclonal antibody (Cat. # MAB3447, 75 μL/well of a 1 μg/mL solution of the antibody in carbonate buffer, pH 9.8). Control wells (also in triplicates) contained 10 μL/well of the same but heat-denatured extracts. The ChAT activity and its protein concentration were normalized to the total protein concentration in each brain extract, and will be referred to as soluble (sChAT), salt-soluble ionic (iChAT), and membrane-bound ChAT (mChAT). In separate untreated wells on the 384-well plate, a serial dilution of choline standard solution was added (the concentration ranged from 50 to 0.39 μM in a twofold dilution series, 50 μL/well, all in triplicates).

After addition of the samples to the wells, 40 μL of cocktail A (see [26]) was added to all wells (with the exception of choline standard), and the plate was sealed and incubated in a moist chamber at 38.5 °C for 60 min under gentle orbital shaking. The plate was then put on ice for 2 min and centrifuged briefly using a plate centrifuge. Then, 25 μL of cocktail B (see [26]) was pipetted to all wells, including the choline standard wells using a multichannel dispenser, and the change in absorbance was monitored every 1–2 min using the kinetic reading option of the spectrophotometer (Tecan Infinite M1000), at 500 nm wavelength. These data were used to calculate the enzyme activity according to the formula presented in [26].

For measuring the ChAT protein level in the sample, the plate was then sealed and incubated overnight at 4 °C. After overnight incubation, the plate was emptied and washed. Then, 50 μL of rabbit-anti-ChAT polyclonal antibody (PAB 14536; Abnova, at a dilution of 1/4000) was added to all wells and the plate was incubated for 1–2 h at 38 °C; then, 25 μL/well of the secondary alkaline phosphatase-conjugated anti-rabbit swine antibody (Cat. # D0306; Dako; at a working dilution of 1/1700, giving a final dilution of ~ 1/5000 in the well) was added. The plate was incubated at RT for 2 h. The plate was washed 4 × 5 min with TBS-T and once with DEA buffer. Then, 75 μL/well of 1 mg/mL solution of the AP substrate, p-nitrophenyl-Na_2_-6H_2_O, in DEA buffer, was added to the wells. The absorbance changes in the wells were monitored either kinetically at 1–3-min intervals at 405 nm wavelength or after a certain incubation time using the endpoint reading option at 405 nm and using 650 nm as reference wavelength. The standard protein was a pre-calibrated pooled plasma sample that had been applied on the plate in a similar manner to the brain homogenate samples. The highest standard of this pooled plasma sample was used at 100× dilution (corresponding to a ChAT protein level of 570 ng/mL), followed by twofold serial dilution points (hence ranging between 570 and 4.75 ng/mL ChAT). It is noteworthy that this ELISA assay works also with recombinant human ChAT (if available) at a concentration range of 500–3.9 ng/mL (twofold dilution series; unpublished observation). Thus, this assay integrates the ChAT activity measurement with determination of ChAT protein levels in the samples, eliminating separate assessments of these two variables, greatly allowing better correlation studies and also saving time and materials.

### NGF Protein Level Measurement

NGF protein level was measured by ELISA using the human β-NGF (Duoset kit, Cat. #DY256, R&D Systems), according to the manufacturer’s protocol with some modifications. Briefly, a 384-well plate (Nunc Maxisorp) was incubated overnight at 4 °C with 50 μL/well of mouse anti-human β-NGF capturing antibody (2.0 μg/mL in carbonate buffer, pH 9.8). The plate was then washed 1 × 5 min with TBS, blocked for 1 h at RT with 5% BSA (prepared in the coating buffer), washed 3 × 5 min with TBS-T (containing 0.01% NaN_3_), and incubated with the standards and samples. As standards, twofold serial dilutions (S_1_–S_14_; S_1_ = 2000 pg/mL) were prepared of recombinant human β-NGF standard in reagent diluent (PBS containing 1% BSA and 0.01% NaN_3_, pH 7.4). Brain homogenates were prepared at 1:2 dilutions in reagent diluent, and 50 μL/well was applied for both samples and standards (all in triplicates) and incubated at 4 °C overnight. Next day, the plate was washed 3 × 5 min with TBS-T and incubated at RT for 3 h with 50 μL/well of the biotinylated goat anti-human β-NGF detecting antibody (kit part #840367; 50 ng/mL prepared in reagent diluent), washed as before, and incubated for 2 h at RT with 50 μL/well of AP-conjugated Streptavidin (Cat. #11093266910, Roche Diagnostics, 1:10,000 dilution prepared in reagent diluent). The plate was washed 2 × 5 min with TBS-T and 1 × 5 min with DEA buffer (1.0 M, pH 9.8), and then incubated with 50 μL/well of 1 mg/mL solution of the AP substrate, p-nitrophenyl-Na_2_-6H_2_O, in DEA buffer. The absorbance changes in the wells at 405 nm wavelength were monitored either kinetically at 1-min intervals or after a certain incubation time and dual reading at 405 and 570 nm as reference wavelength.

### BDNF Protein Level Measurement

For BDNF quantification, brain tissue samples were homogenized in 750 μL of homogenization buffer (85.58 mg/mL sucrose; 50 mM Tris pH 7.5; 25 mM KCl; 0.5 mM PMSF; 1:100 proteinase inhibitor Sigma #P8340; 1:100 phosphatase inhibitor Sigma #P2850; 1:100 phosphatase inhibitor Sigma #P5726; 0.22 μg/μL sodium butyrate) and centrifuged at 4 °C at 9000 rpm for 1 min, in order to obtain the cytosolic soluble fraction (supernatant) that was used for quantification. The BDNF protein level was measured on these samples by ELISA using the BDNF (Human) ELISA Kit (Cat. #KA0329, Abnova), according to the manufacturer’s protocol with minor modifications. Briefly, a pre-coated 96-well plate provided with the kit was incubated with 100 μL/well of standards and samples, in duplicates, at 37 °C for 90 min. As standards, twofold serial dilutions (S_1_–S_7_; S_1_ = 2000 pg/mL; S_7_ = 31.2 pg/mL) of recombinant human BDNF standard were prepared in sample diluent buffer (supplied with the kit). Brain homogenate samples were applied undiluted (undiluted cytosolic fractions), and a buffer blank was also loaded on the plate as a negative control. After incubation, the plate was emptied and subsequently incubated at 37 °C for 60 min with 100 μL/well of biotinylated anti-BDNF antibody (1:100 in the antibody diluent buffer supplied with the kit). The plate was then washed three times with 300 μL/well of 0.01 M TBS pH 7.4, incubated at 37 °C for 30 min with 100 μL/well of avidin-biotin-peroxidase complex (1:100 in ABC diluent buffer supplied with the kit), washed five times as before, and incubated for 25 min at 37 °C with 90 μL/well of the peroxidase substrate TBM, in the dark. Finally, 100 μL/well of TBM stop solution was added to each well to stop the colorimetric reaction and the O.D. was read at 450 nm wavelength, with an Infinite M1000 Tecan microplate reader. BDNF concentration in each well was calculated by comparing their O.D. with the O.D. of BDNF standard wells, by interpolation into a standard curve. BDNF concentration in the samples was subsequently determined by normalizing the BDNF concentration values of each well to the total protein concentration of the corresponding sample, and expressed as picograms per milligram of total protein.

### APP Protein Level Measurement

APP protein level was measured by ELISA using the Human APP Duoset kit (Cat. #DY850, R&D Systems), according to the manufacturer’s protocol with some modifications. Briefly, a 384-well plate (Nunc MaxiSorp) was incubated overnight at RT with 50 μL/well of mouse anti-human APP capturing antibody (4 μg/mL in coating buffer (PBS/0.01% thimerosal, pH 7.4)). The plate was then washed 1 × 5 min with 100 μL/well of PBS/0.01% thimerosal, blocked for 1 h at RT with 100 μL/well of 3% BSA (prepared in the coating buffer), washed 3 × 5 min with 100 μL/well of PBS-T (PBS/0.05% Tween)/0.01% thimerosal, and incubated for 3 h at RT with triplicates of the samples and standards (50 μL/well). As standards, twofold serial dilutions (S_1_–S_8_; S_1_ = 40 ng/mL) of recombinant human APP standard were prepared in 1% BSA/PBS/0.01% thimerosal. Brain homogenate samples were used at the following dilutions in 1% BSA/PBS/0.01% thimerosal: soluble and membrane-bound extracts, 1/15, and ionic extracts, 1/4. After incubation, the plate was washed 5 × 5 min with PBS-T^0.05%^, incubated at RT for 2 h with 50 μL/well of the biotinylated mouse anti-human APP Detection Antibody (300 ng/mL dilution in 1% BSA/PBS/0.01% thimerosal), washed five times as before, and incubated for 1 h at RT with 50 μL/well of alkaline phosphatase (AP)-conjugated streptavidin (Cat. #11093266910, Roche Diagnostics, 1:2000 dilution in 1% BSA/PBS/0.01% thimerosal). The plate was washed 4 × 5 min as before and 1 × 5 min with diethanolamine (DEA) buffer (1.0 M, pH 9.8), and then incubated with 50 μL/well of 1 mg/mL of the AP substrate, p-nitrophenyl-Na_2_-6H_2_O, diluted in DEA buffer. The absorbance changes in the wells at the 405-nm wavelength were monitored with an Infinite M1000 Tecan microplate reader.

### Expression Data Extracted from NCBI GEO (Gene Expression Omnibus) Database

In order to provide proof of concept about the findings of the current study and hypothesis, microarray gene expression data from “Post-mortem Alzheimer’s disease brains: Hisayama Study” available at GEO database were used (reference series GSE36980) [32]. These data have been generated by microarray analysis using the Affymetrix Human Gene 1.0 ST platform from RNA samples (RIN ≧ 6.9) from gray matter of the frontal cortex (*n* = 15 AD, *n* = 18 controls), temporal cortex (*n* = 10 AD, *n* = 19 controls), and hippocampus (*n* = 7 AD, *n* = 10 controls)] of 88 postmortem brains (https://www.ncbi.nlm.nih.gov/sites/GDSbrowser?acc=GDS4758). All AD cases were pathologically diagnosed as AD or AD-like disorder. For this study, gene expression data for the selected genes, studied in the current manuscript, were extracted and used for correlation analysis.

Similarly, another data set from the GEO database (GSE1297) was also considered to confirm the correlation analysis observed using the previous data set [33]**.** This data set contains AD patients of different disease severities (incipient, *n* = 7; moderate, *n* = 8; severe, *n* = 7) and control (*n* = 9) samples, and gene expression microarray was performed from the hippocampal postmortem tissue (https://www.ncbi.nlm.nih.gov/geo/query/acc.cgi?acc=GSE1297). Multiple regression analysis was done using *OMI/HTRA2* and MMSE score as dependent variable, while all other expression data of AD-related genes were used as independent variables.

### Statistical Analyses

Statistical analyses between groups were performed by the nonparametric Mann-Whitney *U* test. The significance levels were set to *p < 0.05. Data are given as mean ± S.E.M. The correlation analyses were done by the nonparametric Spearman rank correlation, which was visualized graphically using a simple regression plot. Multiple regression analysis was used to analyze the GEO data sets.

## Results

### Higher Levels of OMI/HTRA2 Protein in AD than Control Brain

**Fig. 1:**
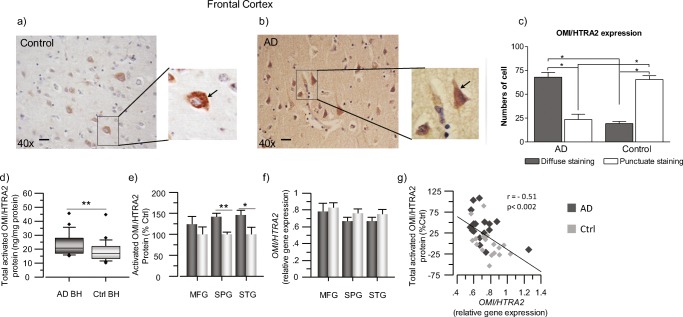
OMI/HTRA2 protein level is higher in AD than control brains. **a**, **b** Immunohistochemical detection of OMI/HTRA2 in human postmortem brain sections of frontal cortex. Representative image of sections from non-demented controls (**a**) and AD (**b**) subjects. Staining: OMI/HTRA2 (brown): anti-OMI/HTRA2 rabbit primary antibody, biotinylated horse anti-rabbit antibody, and ABC-Elite HRP. Counterstaining: hematoxylin (blue). Scale bar = 20 μm. **c** Quantitative analyses showing the number of cells that present either a diffuse or punctuate staining pattern of OMI/HTRA2 in postmortem brain tissues. **d**, **e** Quantification of activated OMI/HTRA2 protein levels in postmortem brain homogenates (BH) of AD subjects (*n* = 6) and non-demented controls (*n* = 6), by sandwich ELISA. **d** Box plot graph showing the overall level of activated OMI/HTRA2 in AD compared to controls, measured in all the extracts from the three brain regions and expressed as ng/mg of total protein. **e** Graph showing the level of activated OMI/HTRA2 in each brain region separately, expressed as percentage of the control levels. **f**, **g***OMI/HTRA2* gene expression was quantified in the postmortem brain samples from the MFG, SPG, and STG brain regions. **f** Graph showing the normalized transcript levels of *OMI/HTRA2* in each of the analyzed brain regions. **g** Negative correlation of *OMI/HTRA2* transcript levels and activated OMI/HTRA2 protein levels, expressed as % of the mean of the controls (Ctrl). Activated OMI/HTRA2 was quantified in medial frontal gyrus (MFG), superior temporalis gyrus (STG), and superior parietal gyrus (SPG) brain regions. The total activated OMI/HTRA2 refers to the sum of its protein levels in all extracts. AD, dark gray squares; non-demented controls, light gray squares. Mean values ± S.E.M. are shown (two asterisks, *p* < 0.01; one asterisk, *p* < 0.05)

Expression of OMI/HTRA2 in human brain tissue was investigated both by immunohistochemistry and by *in-house*-developed sandwich ELISA assay. The demographic characteristics are presented in Table 1. The average age was 80.5 ± 3.5 years for the AD cases and 80.2 ± 3.2 for the control cases (*p* > 0.94), regarding the NBB samples. Similarly, there was no difference in age among the genders, which was equally distributed among the AD and controls. As expected in Alzheimer’s brains, immunohistochemical analyses showed a robust reduction in the number of neurons in the AD compared to the control brains. Overall, the immunostaining analyses indicated that the neuronal OMI/HTRA2 staining was more pronounced in the frontal cortex of the AD compared to the control brain. The immunostaining of OMI/HTRA2 exhibited two distinct staining patterns in the neurons of the frontal cortex of the AD compared to control brains: some neurons showed punctuate staining and others a diffuse cytosolic staining, which may reflect the release of OMI/HTRA2 into the cytosol of the neurons (Fig. 1a, b). Quantitative analysis revealed a significant increase of diffuse cytosolic staining of OMI/HTRA2 in the frontal cortex of the AD compared to control brains, indicating that the release of active OMI/HTRA2 into the cytosol can be involved in initiation of cell death in the AD brain (Fig. 1c).

### The Level of Activated OMI/HTRA2 Protein in Different AD Brain Regions

In order to quantify the protein expression of active OMI/HTRA2 in different brain regions, we established an in-house optimized sandwich ELISA, specific for the activated form of OMI/HTRA2 (Fig. S1). To get some insights, we prepared three consecutive protein extracts, namely soluble, ionic, and membrane extracts, from MFG, STG, and SPG brain regions, and the level of the activated form of OMI/HTRA2 in each extract was quantified by the in-house-developed ELISA assay.

The overall amount of activated form of OMI/HTRA2 in AD and control brains is displayed and compared in Fig. 1d, which shows a significant increase in activated OMI/HTRA2 levels in the AD brain.

The protein level of activated OMI/HTRA2 was significantly increased in SPG (42%, *p* < 0.003) and STG (48%, *p* < 0.05) but not in MFG brain regions of the AD brains (Fig. 1e). Then, we also compared the levels of activated OMI/HTRA2 in each different brain extract. These analyses are shown in supplementary Fig. S2 and indicated that activated OMI/HTRA2 was predominantly extracted in the high-salt-containing extraction buffer (*i-*), followed by the soluble (*s-*) and membrane (*m-*) extracts (*i-*OMI/HTRA2>> *s-*OMI/HTRA2>> *m-*OMI/HTRA2). The levels of these extracted forms of OMI/HTRA2 protein were analyzed and compared in the MFG, SPG, and STG regions of the AD and control brains.

The level of *s-*OMI/HTRA2 protein was 81% higher in the SPG region of the AD brain compared to the controls (*p* < 0.003, Fig. S2a), while it was just numerically higher in the AD MFG and STG brain regions (10–25%, *p* > 0.05). The levels of *i*-OMI/HTRA2 protein were also just numerically higher in the MFG, SPG, and STG regions of the AD brain compared to the controls (12–39%, all *p* > 0.13; Fig. S2b). The protein level of *m-*OMI/HTRA2 was 48% higher in the AD MFG region compared to the control (*p* < 0.06, Fig. S2c), and was numerically, 8 and 34%, higher in SPG and STG regions of the AD brain compared to control (respectively; *p* > 0.24).

### *OMI/HTRA2* Gene Expression Level Correlated Negatively with Activated OMI/HTRA2 Protein Level in Different Regions of AD and Control Brains

Next, we looked at the *OMI/HTRA2* gene expression that was analyzed and quantified in the three different brain regions of the AD and control brains. The demographic characteristics are presented in Table 1. In contrast to the quantified protein levels of activated OMI/HTRA2, the gene expression analyses indicated a mild to moderate reduction in the *OMI/HTRA2* transcripts in the AD brains compared to controls (Fig. 1f). Correlation analyses revealed a significant negative correlation between *OMI/HTRA2* gene expression and active OMI/HTRA2 protein level in the brain homogenates (Fig. 1g).

### Correlation of OMI/HTRA2 Activated Protein and Gene Expression with Cholinoceptive Markers

Next, the *OMI/HTRA2* gene and protein expression levels were correlated with levels of cholinergic markers in the AD-affected and control brains.

**Fig. 2:**
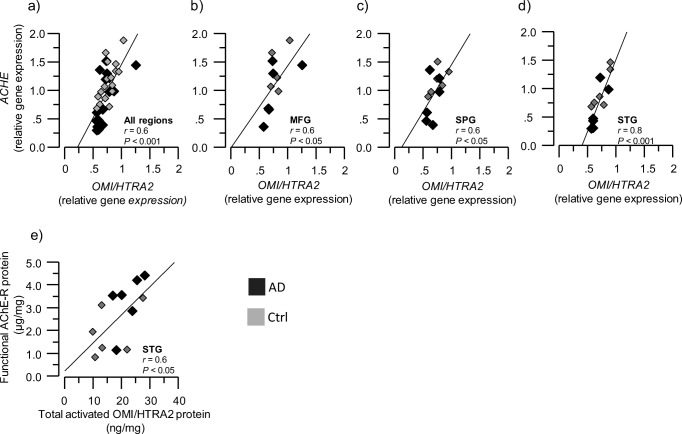
Positive correlation between *OMI/HTRA2* and *ACHE* gene and protein expression. *OMI/HTRA2* and *ACHE* gene and protein expression were quantified in AD subjects’ (*n* = 6) and non-demented controls’ (*n* = 6) postmortem brain, in medial frontal gyrus (MFG), superior temporalis gyrus (STG), and superior parietal gyrus (SPG) brain regions. **a**–**d** Graphs showing the positive correlation between *OMI/HTRA2* and *ACHE* gene expression, in the average of the three analyzed brain regions (**a**) and in each region separately (**b–d**); **e** graph showing the positive correlation between activated OMI/HTRA2 protein level and AChE-R protein level in the STG region. *ACHE* = acetylcholinesterase gene; AChE-R = “Read-through” AChE splice variant protein

Cholinergic signaling is most likely dependent on several enzymes that are either involved in ACh synthesis (ChAT) or degradation (AChE and BuChE). We therefore performed correlative analyses between *ACHE* and *OMI/HTRA2* gene and protein expression in brain homogenates from AD and controls in the MFG, SPG, and STG regions. Correlation analyses indicated a positive association between *ACHE* and *OMI/HTRA2* gene expression in the overall and in each of the three brain regions (Fig. 2a–d). The read-through AChE protein variant (AChE-R) is regarded as a soluble variant of AChE that can be present both intracellularly and extrasynaptically, and is closely associated with various stressful stimuli so that it is often referred to as the stress-associated AChE-R variant. In the mouse brain, for instance, AChE-R is persistently overexpressed after trauma and is implicated in stress-induced changes in neuronal structure and function [34, 35]. Here, we found a positive correlation between total protein expression of AChE-R and total activated OMI/HTRA2 expression in the STG brain region (Fig. 2e).

**Fig. 3:**
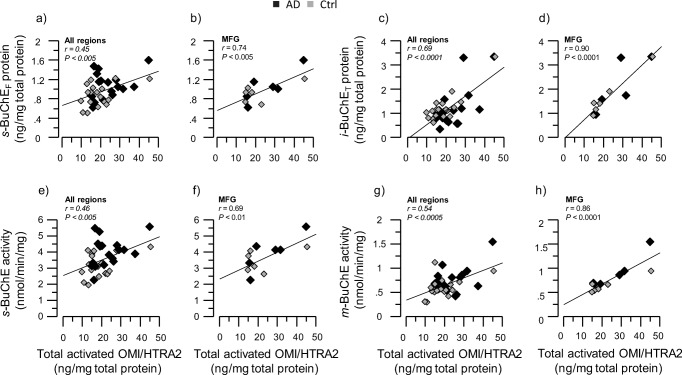
Positive correlation of activated OMI/HTRA2 with BuChE protein expression and activity. **a**–**d** Graphs showing the positive correlation of OMI/HTRA2 with *s-*BuChE (**a**, **b**) and *i-*BuChE (**c**, **d**) protein expression in the average of the three analyzed brain regions (**a**, **c**) and in the MFG region (**b**, **d**). **e**–**h** Graphs showing the positive correlation of OMI/HTRA2 with *s-*BuChE (**e**, **f**) and *m-*BuChE (**g**, **h**) protein activity in the average of the three analyzed brain regions (**e**, **g**) and in the MFG region (**f**, **h**). BuChE = butyrylcholinesterase. BuChE protein expression and activity were quantified in the brain tissue extract from AD (*n* = 6) and non-demented controls (*n* = 6). Postmortem brain tissues were from medial frontal gyrus (MFG), superior temporalis gyrus (STG), and superior parietal gyrus (SPG) regions. *s-*, *m-*, and *i-* refer to amount of BuChE quantified in the soluble (*s-*), ionic (*i-*), and membrane-bound (*m-*) protein extracts from each of the brain tissues and regions as described in the “Materials and Methods” section

Another soluble ACh-degrading enzyme is BuChE, which is closely linked to astroglial function and immunoregulatory processes [30, 36, 37]. We found no correlation between the *BCHE* and *OMI/HTRA2* gene expression (data not shown). However, the BuChE protein levels in the soluble and ionic brain extracts showed positive correlation with the total activated OMI/HTRA2 protein (Fig. 3a–d) in the average of the three brain regions and particularly in the MFG brain region. Moreover, soluble and membrane-bound BuChE enzymatic activity was positively correlated with the activated OMI/HTRA2 total protein expression in the average of the three brain region extracts and more specifically in the MFG region (Fig. 3e, f) (*r* = 0.69, *p* < 0.01; *r* = 0.86, *p* < 0.0001).

**Fig. 4:**
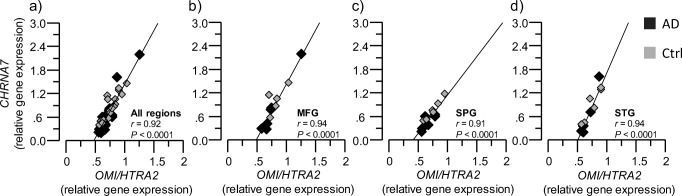
Positive correlation between *OMI/HTRA2* and *CHRNA7* gene expression. *OMI/HTRA2* and *CHRNA7* gene expression were quantified in AD subjects’ (*n* = 6) and non-demented controls’ (*n* = 6) postmortem brain, in medial frontal gyrus (MFG), superior temporalis gyrus (STG), and superior parietal gyrus (SPG) brain regions. **a**–**d** Graphs showing the positive correlation between *OMI/HTRA2* gene expression and *CHRNA7* gene expression, in the average of the three analyzed brain regions (**a**) and in each region separately (**b**–**d**). AD, dark gray squares; non-demented controls, light gray squares. *CHRNA7* = α7 nicotinic acetylcholine receptor gene

Cholinergic signaling is mediated through cholinergic receptors, i.e., muscarinic and nicotinic acetylcholine receptors (AChRs). The α7 nicotinic AChRs (α7nAChRs) are of particular relevance and importance for inflammatory processes, and for the function of glial-neuronal interfaces in the brain [27, 38]. Intriguingly, there is evidence of presence of mitochondrial nicotinic receptors, which seem to be involved in protection against apoptosis [39]. We hence examined the relation between *OMI/HTRA2* and α7nAChRs (*CHRNA7*) gene expression, which revealed strong positive correlation in all three brain regions (Fig. 4a–d). We did not measure the protein levels of α7nAChRs in the brain homogenate extracts.

### High Correlation of OMI/HTRA2 with the Cholinergic Marker ChAT

**Fig. 5:**
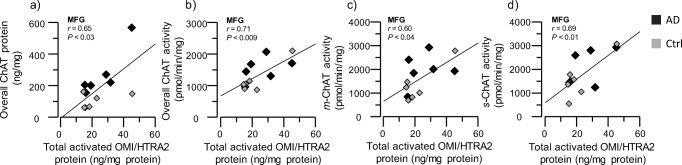
Positive correlation of activated OMI/HTRA2 with ChAT protein expression and activity. ChAT protein expression and activity were quantified in AD subjects’ (*n* = 6) and non-demented controls’ (*n* = 6) postmortem brain medial frontal gyrus (MFG), superior temporalis gyrus (STG), and superior parietal gyrus (SPG) regions, within the soluble (*s*), ionic (*i*), and membrane-bound (*m*) protein extracts, and then correlated to the activated OMI/HTRA2 levels in the same regions. **a** Graph showing the positive correlation of OMI/HTRA2 level and total ChAT protein level in the MFG brain region. **b**–**d** Graphs showing the positive correlation of OMI/HTRA2 level with the total ChAT (**b**), *m-*ChAT (**c**), and *s-*ChAT (**d**) enzymatic activity in the MFG brain region. ChAT = choline acetyltransferase

The signature of cholinergic neurons/cells is the expression of the core ACh-synthesizing enzyme, ChAT. We hence determined the protein level and activity of ChAT in the brain extracts and examined whether any relationship between ChAT and OMI/HTRA2 levels existed. The overall pattern of these correlation analyses indicated high positive correlation between activated OMI/HTRA2 and both ChAT protein levels and ChAT activity in various brain extracts, in particular from the MFG brain region (Fig. 5a–d).

### *NGF* and *BDNF* Gene Expression Levels Positively Correlated with *OMI/HTRA2* Gene Expression

Nerve growth factor (NGF) and brain-derived neurotrophic factor (BDNF) promote growth, development, regulation, differentiation, and survival of peripheral and central nervous system neurons, in particular forebrain cholinergic neurons [25, 40]. We therefore investigated whether a relationship exists between the gene and protein expression of OMI/HTRA2, with NGF and BDNF.

**Fig. 6:**
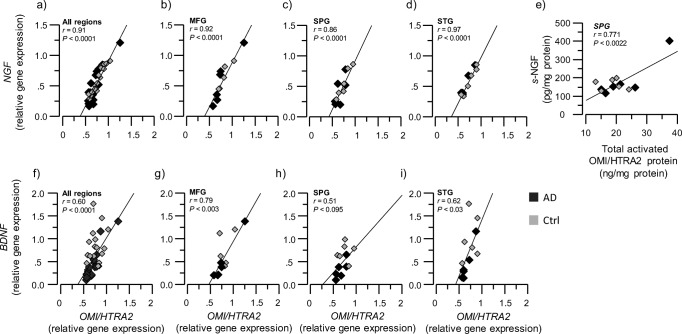
Positive correlation between OMI/HTRA2 and neurotrophic factors at both the gene expression and protein levels. OMI/HTRA2, NGF, and BDNF gene expression and protein levels were quantified in AD subjects’ (*n* = 6) and non-demented controls’ (*n* = 6) postmortem brain, in medial frontal gyrus (MFG), superior temporalis gyrus (STG), and superior parietal gyrus (SPG) brain regions. **a**–**d** Graphs showing the positive correlation between *OMI/HTRA2* gene expression and *NGF* gene expression, in the average of the three analyzed brain regions (**a**) and in each region separately (**b**–**d**). **e** Graph showing the positive correlation between *s-*NGF and activated OMI/HTRA2 protein levels in the SPG region. **f**–**i** Graphs showing the positive correlation between *OMI/HTRA2* gene expression and *BDNF* gene expression, in the average of the three analyzed brain regions (**f**) and in each region separately (**g**–**i**). NGF = nerve growth factor, BDNF = brain-derived neurotrophic factor, *s-*NGF = amount of NGF measured in the soluble brain extract

*NGF* gene expression in all three brain regions exhibited high correlation with *OMI/HTRA2* gene expression (Fig. 6a–d). Similarly, *OMI/HTRA2* and *BDNF* gene expression were correlated (Fig. 6f–i).

We also measured the protein levels of NGF and BDNF in the brain extracts. In concordance with gene expression analyses, there was a positive correlation between activated OMI/HTRA2 total protein expression and NGF, in particular between activated OMI/HTRA2 and soluble *s-*NGF in the SPG region (*r* = 0.77, *p* < 0.01; Fig. 6e). However, no correlation between BDNF and OMI/HTRA2 was observed.

### *OMI/HTRA2* Gene Expression in Three Brain Regions Showed High Correlation with *APP* and *MAPT* Gene Expression

Two major hallmarks of AD pathology, namely the Aβ peptide and tau aggregates, result from the protein products of *APP* and *MAPT* genes. Therefore, we investigated whether an association between *APP* or *MAPT* and *OMI/HTRA2* gene and protein expression existed in the brains of AD and controls.

**Fig. 7:**
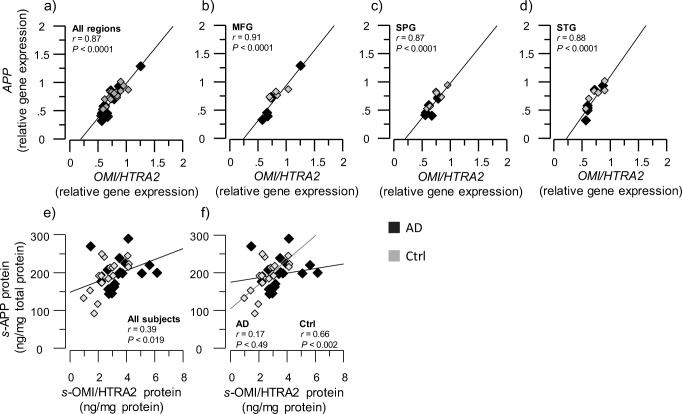
Positive correlation between *OMI/HTRA2* and *APP* at both the gene expression and protein levels. *OMI/HTRA2* and *APP* gene expression, and their protein levels within the soluble (s) protein extracts, were quantified in AD subjects’ (*n* = 6) and non-demented controls’ (*n* = 6) postmortem brain, in medial frontal gyrus (MFG), superior temporalis gyrus (STG), and superior parietal gyrus (SPG) brain regions. **a**–**d** Graphs showing the positive correlation between *OMI/HTRA2* gene expression and *APP* gene expression, in the average of the three analyzed brain regions (**a**) and in each region separately (**b**–**d**)**. e**, **f** Graphs showing the positive correlation of *s-*OMI/HTRA2 with *s-*APP protein expression in the average of the three analyzed brain regions, in all subjects (**e**) and in AD versus controls (**f**). APP = amyloid-beta precursor protein, *s-*APP and *s-*OMI/HTRA2 refer to amount of APP and activated OMI/HTRA2 proteins measured in the soluble brain extracts as described in the “Materials and Methods” section

We found a positive correlation between *OMI/HTRA2* and *APP* gene expressions in all brain regions (all *r* values > 0.8, *p* < 0.001, Fig. 7a–d). The protein level of soluble APP (*s-*APP) correlated with the soluble activated OMI/HTRA2 protein (*s-*OMI/HTRA2) but only in the brain regions of control brain homogenates (*r* = 0.66, *p* < 0.002, Fig. 7e, f).

**Fig. 8:**
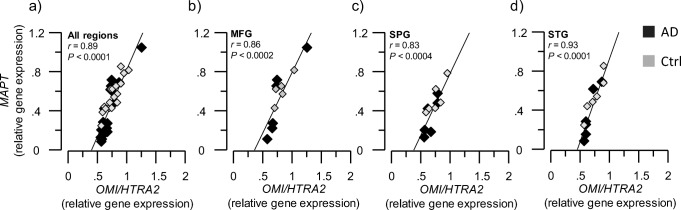
Positive correlation of *OMI/HTRA2* and *MAPT* gene expression. *OMI/HTRA2* and *MAPT* gene expression were quantified in AD subjects’ (*n* = 6) and non-demented controls’ (*n* = 6) postmortem brain, in medial frontal gyrus (MFG), superior temporalis gyrus (STG), and superior parietal gyrus (SPG) brain regions. **a**–**d** Graphs are showing the positive correlation between *OMI/HTRA2* gene expression and *MAPT* gene expression, in the average of the three analyzed brain regions (**a**) and in each region separately (**b**–**d**). MAPT = microtubule-associated protein tau

Similarly, *MAPT* gene expression exhibited positive correlation with *OMI/HTRA2* gene transcript levels in all the examined brain regions (all *r* values > 0.8, *p* < 0.001, Fig. 8a–d). The tau (MAPT) protein level was not measured; hence, no correlation analyses could be performed.

### Gene Expression Data from GEO Database Support the Correlation Analyses Between *OMI/HTRA2* and Cholinergic Markers

**Fig. 9:**
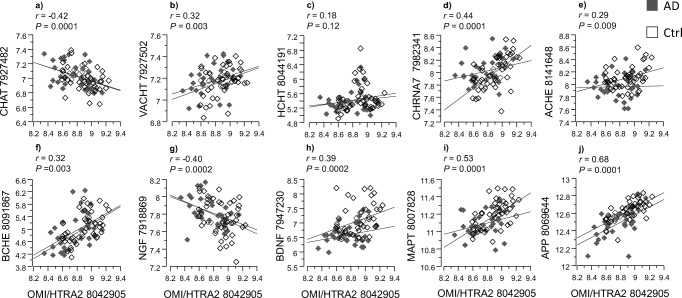
Independent correlation analyses between OMI/HTRA2, cholinergic markers, and AD-related gene expression. In order to provide proof of concept about our hypothesis, correlation analyses were done using microarray data from human postmortem brain tissue deposited in the Geo database. The microarray gene expression data were from “Post-mortem Alzheimer’s disease brains: Hisayama Study” (reference series GSE36980) [32]. **a**–**f** illustrate the correlation between *OMI/HTRA2* gene expression and the gene expression of *CHAT*, *VACHT*, *HCHT*, *CHRNA7 ACHE*, and *BCHE*, respectively. **g**, **h** show correlation between *OMI/HTRA2* gene expression and the gene expression of the neurotrophic factors, *NGF* and *BDNF*, respectively. **i**, **j** are correlation between the gene expression of *OMI/HTRA2* and the AD-associated marker tau (*MAPT* gene) and the amyloid precursor protein (*APP gene*). The microarray data were from gray matter tissues of frontal cortex (*n* = 15 AD; *n* = 18 controls), temporal cortex (*n* = 10 AD, *n* = 19 controls), and hippocampus (*n* = 7 AD, *n* = 10 controls) of 88 postmortem brains (https://www.ncbi.nlm.nih.gov/sites/GDSbrowser?acc=GDS4758)

Admittedly, the number of postmortem brain tissues were limited in the current study; therefore, to ascertain the correlation findings, we performed similar analyses with an independent set of gene expression data from the GEO database repository [32]. The result of this analysis is shown in Fig. 9.

In agreement with our current correlation studies, the correlation analyses using data set, GSE36980, from the GEO database confirmed that *OMI/HTRA2* gene expression correlated with the gene expression of the cholinergic markers *CHRNA7*, *ACHE*, and *BCHE* (Fig. 9d–f). The gene expression data for *CHAT*, vesicular ACh transporter (*VACHT*), and high-affinity choline transporter (*HCHT*), which were not determined in our study, were available in the GEO bank data set. The *OMI/HTRA2* gene expression also correlated with these additional cholinergic genes transcripts (Fig. 9a–c), providing additional support to the findings in the current study.

Similar to our data set, the *OMI/HTRA2* gene expression correlated with the gene expression of *NGF*, *BDNF*, *MAPT*, and *APP* in the GSE36980 data set (Fig. 9g–j). However, it should be noted that while *OMI/HTRA2* and *NGF* gene expression showed positive correlation in our data set, they showed inverse correlation in this GEO data set. The mode of correlation between the rest of the variables was otherwise similar to our data set.

We also used a second data set (GSE1297) from the GEO database and performed multiple regression analysis. Importantly, this data set also contained a measure of global cognition, assessed by MMSE. We hence used first MMSE as a main dependent outcome that also reflects the severity of the disease (Supplementary Table S1). The multiple regression analyses revealed an overall highly significant correlation (*r* = 0.84 and *p* < 0.0006). The variable with partial contributions were *OMI/HTRA2* (*r* = 0.84, *p* = 0.0004), *CHAT* (*r* = 0.60, *p* = 0.001), *ACHE* (*r* = − 0.42, *p* ≤ 0.014), *CHRNA7* (*r* = 0.35, *p* = 0.03), *APP* (*r* = 0.48, *p* ≤ 0.013), and *MAPT* (*r* = 0.50, *p* = 0.003).

Next, we used as before the expression of Omi/HTRA2 as the dependent variable as it is the focus of this study (overall *r* = 0.82, *p* < 0.0001, Supplementary Table S2). The result showed significant association of *OMI/HTRA2* with *CHAT* (*r* = − 0.37, *p* = 0.003), *ACHE* (*r* = 0.65, *p* ≤ 0.0001), and *APP* (*r* = − 0.46, *p* = < 0.0004), respectively. There were no significant contribution of sex and age in these analyses.

## Discussion

**Fig. 10:**
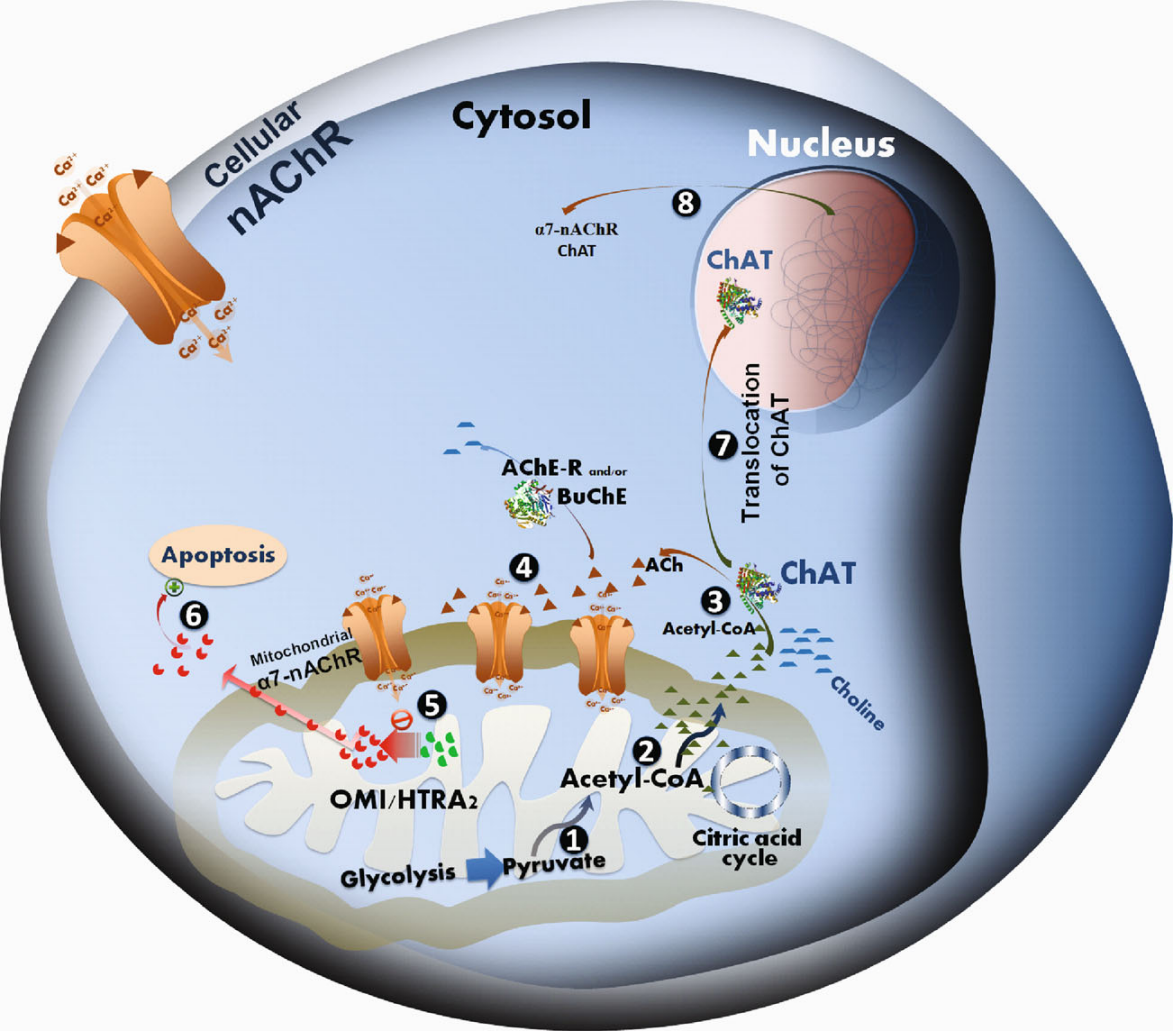
Hypothetical pathway linking the mitochondrial function, OMI/HTRA2, apoptosis, and intracellular cholinergic signaling. The well-documented presence of nicotinic acetylcholine receptors (nAChRs) at the mitochondrial outer membrane defines all eukaryotic cells as cholinoceptive cells and acetylcholine, the main known endogenous ligand of these receptors, as a universal intracellular signaling molecule. (1) As a matter of fact, biosynthesis of ACh is directly linked to mitochondrial glycolysis and citric acid cycle. In mitochondria, glucose converted to pyruvate, and thereby production of acetyl-Coenzyme A (*A-CoA*) [41], (2) which is partly used in the citric acid cycle for generation of bioenergetic molecules such as ATP, and (3) the rest may enter into the cytoplasm for instance for biosynthesis of ACh by ChAT, which is apparently required for activation of the mitochondrial nAChR (*mt-*nAChR). (4) The mt-nAChRs and their regular activation by intracellular ACh may hence function as a biodynamic sensor. For instance, in favorable conditions, a high access to glucose is sensed due to production of ACh and activation of *mt-*nAChRs, (5) thereby preventing auto processing of the mitochondrial serine protease OMI/HTRA2, (6) which could otherwise lead to activation of intrinsic mitochondrial apoptotic pathway [39, 42]. Certain conditions, such as ongoing pathological processes (like those occurring in the AD brain) or other stressful stimuli, may either cause mitochondrial dysfunction and/or greatly limit mitochondrial access to glucose and production of pyruvate, leading to diminished synthesis of *A-CoA* and thereby ACh. Indeed, there are direct links between ACh and insulin secretion [43], and/or insulin resistance and Alzheimer’s disease [44], glucose metabolism [45], as well as mitochondrial dysfunction [46]. In such circumstances, a hypothetical pathway is that as a mean to maintain protective action of ACh through *mt-*nAChRs [39, 42], the reduced cytosolic *A-CoA* induces (7) translocation of ChAT into the cell nucleus, where ChAT somehow will participate in modulation of gene expression of various key proteins [47]. (8) Two of these key proteins are most likely nAChR and ChAT itself. This could explain the positive correlation between OMI/HTRA2, nAChR, and ChAT reported in the current study (Figs. 4 and 5). In other words, an increase in the nAChR levels will enhance the possibility of activation of the receptors even at reduced levels of intracellular ACh, while an increase in ChAT levels could reflect an attempt to increase ACh biosynthesis. This may work if the cause is not a reduced access to pyruvate, and thereby a severe depletion of *A-CoA*, but rather excessive degradation of ACh. This latter scenario may be caused, for instance, by certain stress stimuli, known to shift the *ACHE* gene transcription in favor of the soluble stress-associated read-through AChE splice variant (AChE-R) [48–50], which could result in excessive degradation of the intracellular ACh. The observed positive correlation between OMI/HTRA2 and the stress-associated AChE-R variant support this latter notion. However, when such preventive measures (totally or partially) fail to normalize the intracellular ACh-signaling, OMI/HTRA2 mitochondrial serine protease (perhaps together with cytochrome C) is expected to be released into the cytoplasm, initiating apoptosis cascades [6]. This explains our finding of higher levels of activated OMI/HTRA2, and its cytoplasmic dispersion in the AD brain compared to control (Fig. 1). The association of OMI/HTRA2 with BuChE may be related to the same phenomenon occurring in non-neuronal cells, such as astrocytes, in which the intracellular BuChE may replace the AChE-R variant since BuChE in the brain is known to have mainly astroglial origin

Few reports on gene and protein expression of OMI/HTRA2 in the AD brain exist, in particular in relation to markers of the main neuronal dysfunction in AD, namely the cholinergic deficit. We report a higher level of activated OMI/HTRA2 in three different brain regions, as well as a different intracellular distribution of OMI/HTRA2 in frontal cortex sections of the AD brain compared to control. Moreover, we show a coherent pattern of relationship between the OMI/HTRA2 gene and protein expression, cholinergic biomarkers together with other disease-specific pathophysiological markers (Fig. 10). This was supported by similar analyses on two independent data sets of gene expression from GEO database. In particular, one of the data sets contained the measure of global cognition, MMSE, which showed high correlation with the majority of genes that were the focus of this study.

In a previous report, we used a semi-quantitative western-blotting analysis of OMI/HTRA2 protein levels in the AD brain [21]. Here, in addition to performing immunohistochemical analyses of the OMI/HTRA2 localization pattern on brain sections, we measured the activated OMI/HTRA2 protein by a quantitative assay in three types of brain extracts from three different brain regions of AD and control subjects. It is noteworthy that the current study provided a new sensitive and quantitative method for accurate measurement of the processed form of OMI/HTRA2 protein that can be a useful research tool in conducting future studies.

The punctuate pattern of OMI/HTRA2 protein immunostaining in neurons in the control brain was in agreement with its putative localization into the mitochondria. In the AD brain, however, OMI/HTRA2 showed a diffuse staining pattern in the neuronal cytoplasm. This might indicate release of activated OMI/HTRA2 into the cytoplasm of neurons in the AD brain. Indeed, it is shown that certain types of stimuli result in intra-mitochondrial processing of OMI/HTRA2, leading to proteolytic activation and the release of OMI/HTRA2 into the cytosol [19]. This relocalization of OMI/HTRA2 is in turn expected to produce a diffuse staining pattern, observed in histochemical analyses [19]. Thus, the cytoplasmic detection of OMI/HTRA2 may reflect a high proteolytic activity of OMI/HTRA2 in the AD brain compared to control, in agreement with our previous report [21].

A common key feature of the major dementia disorders, such as AD, Lewy body dementia, and Down’s syndrome, is a selective vulnerability of the central cholinergic neuronal system to AD-type pathological events [51, 52]. However, the underlying molecular mechanisms remain largely unknown. In this study, we found that activated OMI/HTRA2 protein expression was increased in the brains affected by AD, and this correlated positively with the level of the core-cholinergic enzyme, ChAT, as well as with the level of the stress-associated ACh-degrading enzyme AChE-R in some of the examined brain regions. To our knowledge, this is the first report that shows correlation between OMI/HTRA2 expression and these cholinergic markers. This positive association is, however, unexpected since OMI/HTRA2 release is generally linked to apoptosis or cell death [53] and hence is expected to show inverse association with the levels of ChAT, as the cholinergic neurons degenerate in the AD brain. In addition, OMI/HTRA2 also showed positive association with the ACh-degrading capacity in the brain.

A hypothetical explanation for these seemingly paradoxical observations might reside in a role of cholinergic signaling in mitochondrial functions, as outlined in Fig. 10. Numerous studies report the presence of nicotinic AChRs, in particular α7nAChR in the outer membrane of mitochondria [54]. Both cell membrane (*cm-*) and mitochondrial (*mt-*) nAChRs seem to be involved in the protection and/or regulation of mitochondrial function during cell proliferation, survival, and death processes [39, 54–56]. Upon stressful stimuli, the *ACHE* gene expression is shifted towards protein expression of the soluble AChE-R splice variant, which is also known to have certain intra-neuronal function [57]. Accumulation of intra-neuronal AChE-R is then expected to greatly reduce the level of intra-neuronal ACh, which is supposed to exert a tunic action on *mt-*nAChRs, and thereby mediate protective/regulatory feedback signaling about the surrounding (intra- and extracellular) microenvironment to mitochondria. Indeed, evidence suggests that nicotine relieves anxiogenic-like behavior in mice that overexpress the read-through AChE-R [58]. Reduced levels of ACh may hence gradually lead to OMI/HTRA2 processing and release from mitochondria. This might be related to regulation of the intracellular Ca^2+^ levels in mitochondria, opening of the permeability transition pore machinery leading to the release of cytochrome c and/or OMI/HTRA2 [56, 59].

Alternatively or additionally, various stressors cause hyperexcitation of the cholinergic system and alter the *ACHE* gene expression [49], switching *ACHE* mRNA splicing towards the production of the AChE-R variant [60]. Similarly, OMI/HTRA2 activation is caused by a variety of stress conditions including heat-shock or tunicamycin treatment [61, 62]. This OMI/HTRA2 activation has been suggested to serve as a modulator of stress responses [63], and neuron-specific over-expression of OMI/HTRA2 may hence protect against stress [12]. Thereby, the positive correlations between OMI/HTRA2 and AChE-R in the current report are in line with the previous reports. This positive correlation in turn may indicate a close mechanistic link between these two stress-related proteins, OMI/HTRA2 and AChE-R, since an increase in OMI/HTRA2 protease activity is reported after stress condition in differentiated neuroblastoma cells [19] and in the AD brain [21]. Of note, AChE-R exerts also a neuroprotective effect [64]. The positive correlation may also result from the feedback mechanism where OMI/HTRA2 is increased in AD.

A third alternative scenario may be a pathomechanism initiated through abnormal mitochondrial function, in particular in cholinergic neurons/cells (Fig. 10). Cytosolic ACh biosynthesis by ChAT requires equimolar amount of acetyl-coenzyme A, a high energetic cofactor that is produced by mitochondria from pyruvate during glycolysis. Malfunctioning mitochondria and/or low glucose levels may hence result in reduced access to acetyl-CoA, and thereby reduced ACh biosynthesis by ChAT. This might activate an intra-nuclear feedback signaling, by translocation of ChAT to the nucleus, triggering an increased expression of ChAT in an attempt to recover ACh levels, and/or to increase expression and protein levels of *mt-*nAChRs to compensate for reduced ACh levels. This is in line with at least three independently reported phenomena: (i) ChAT is one of the few enzymes known to possess a splice variant with a nuclear localization signaling motif at its amino terminus [65], allowing it to enter the nucleus on demand; (ii) ChAT expression/activity seems to increase in the brain of patients at an early stage of Alzheimer’s disease, namely mild cognitive impairment [66], contrasting its severe reduction in the brain at later stages of AD; (iii) mitochondrial dysfunction is reported as one of the features of AD [67, 68]. Thus, the positive correlation that was observed here between levels of ChAT, nAChRs, and OMI/HTRA2 may represent an ongoing feedback process in normalizing the level of intracellular (and extracellular) ACh. This might also explain the positive associations between the growth factors (NGF and BDNF) and OMI/HTRA2, since cholinergic neurons are known to be dependent on NGF signaling.

In these contexts, the observed strong positive association between OMI/HTRA2 and APP gene and protein expression is in line with what might be expected in the brain of AD patients. This may therefore reflect the mutual links between APP processing and cholinergic deficit in AD [69, 70] and/or the nuclear form of ChAT [47]. In addition, a recent report directly links the Aβ peptides’ effect on acetylcholine homeostasis through formation of reactive ACh-degrading complexes with cholinesterases [71]. Furthermore, another report shows a preferential intra-neuronal accumulation of Aβ peptides in cholinergic neurons in the human brain regardless of the age, and disease status [72], emphasizing a biological role for Aβ production and release in the function of the cholinergic system. Thus, the release of OMI/HTRA2 may be a consequence of a dysfunctional cross-talk between APP cleavage, production of Aβ peptides, and cholinergic signaling. There are also other reports that link OMI/HTRA2 directly to cleavage of APP in mitochondria [73] and/or to a regulatory function of APP metabolism through ER-associated degradation [74].

This study has several limitations. Due to limited availability of brain tissue, we were able to perform only immunohistochemical analyses with regard to OMI/HTRA2 expression in the brain sections. Similarly, there was not enough brain homogenate available to measure levels of *MAPT* gene product (tau protein), as well as of α7nAChRs protein in the brain. Furthermore, this study had an explorative nature and thereby some of the interpretations should be regarded as speculative or hypothesis generating. Admittedly, a number of findings were based on correlation analysis, and hence, further studies are required to substantiate the findings. Nonetheless, we were able to substantiate the major findings by performing similar correlation analyses on two completely independent gene expression data sets from GEO database, thereby warranting further investigation on the outlined hypothesis. For instance, in vitro pharmacological investigation in primary neuronal culture by using nicotinic agonists or cholinesterase inhibitors should provide insight about the role of intracellular ACh on decreasing OMI/HTRA2 expression and level of its activated form. Pharmacological study by an inhibitor of ChAT is instead expected to increase the expression, production, and release of OMI/HTRA2 into cytosol. It should be also feasible to study the effect of such drugs on translocation of the nuclear form of ChAT from cytosol to the nucleus.

## Concluding Remarks

*In conclusion*, the current results demonstrate higher levels of the mitochondrial serine protease OMI/HTRA2 in the AD brain and a coherent pattern of correlations between the activated form of OMI/HTRA2 and several key proteins and biomarkers involved in the AD pathology, warranting further studies to explore these observations in search for new therapeutic windows for treatment of this devastating disease. This study shows that the release of OMI/HTRA2 in the cytosol may be intimately linked to cholinergic signaling and may serve as a stress response in neurodegenerative conditions like AD.

## Electronic supplementary material

Fig. S1 Assay controls for the in-house developed sandwich ELISA specific for the activated form of OMI/HTRA2. A sandwich ELISA assay was setup and used to quantify the protein level of activated OMI/HTRA2 in the brain extracts from three different brain regions of patients and controls. OMI/HTRA2 protein was adsorbed to the wells of an ELISA plate, pre-coated by the capturing antibody, rabbit polyclonal anti-OMI/HTRA2 antibody (AF1458; R&D systems). This antibody detects both the precursor (unprocessed, 49-50 kDa) and the processed (activated, ~36 kDa) bands of OMI/HTRA2 protein under reducing conditions of western blotting (**a**). Then the mouse monoclonal anti-OMI/HTRA2 antibody (MAB1458, R&D System) was used, which only detects the activated form of the OMI/HTRA2 protein (**b**). A comparison of blot **a,** and **b** suggests that the detecting antibody requires epitope exposure available in the processed form of OMI/HTRA2 (activated form). The graph in (**c**) illustrate a representative standard curve of a serial dilution of recombinant human OMI/HTRA2 protein (1458-HT; R&D systems) used in the in-house ELISA to quantify the activated form of OMI/HTRA2 ELISA assay. In a typical assay setup, the concentration of *rh*OMI/HTRA2 standard protein ranged between 125 ng/mL to 0.031 ng/mL, and the optical density of OMI/HTRA2 protein in the brain extracts were in the middle of the standard curve. Additional details are described in the Material and Method section. The western blots were obtained from R&D System web-site with their permission. (PNG 360 kb)

High resolution image (EPS 1173 kb)

Fig. S2 Levels of activated OMI/HTRA2 in different brain extracts. Three different brain extracts were consecutively prepared using different homogenization buffers to extract soluble (*s*), ionic (*i*) and membrane bound (*m*) proteins. The extracts were prepared from three brain regions, namely from MFG (medial frontal gyrus), STG (superior temporalis gyrus) and SPG (superior parietal gyrus) regions. Post-mortem brain tissues were from six AD and six non-demented controls. Activated OMI/HTRA2 protein levels were quantified and defined as soluble (s), ionic (i), and membrane-bound (m) OMI/HTRA2 protein extracts), by an in house sandwich ELISA as described in the “Material and Method” section (graphs **a**, **b**, **c**, respectively). All values are normalized to the total protein in the extracts and are expressed as ng of OMI/HTRA2 protein/mg of total protein. Noteworthy, the absolute majority of activated OMI/HTRA2 protein was found in *ionic* brain extract. **p <0.01;^*#*^*p <0.06*. (PNG 126 kb)

High resolution image (EPS 934 kb)

ESM 2 (DOCX 17 kb)
