# Supplementary figures and images for: Increased Active OMI/HTRA2 Serine Protease Displays a Positive Correlation with Cholinergic Alterations in the Alzheimer’s Disease Brain
Source: Mol Neurobiol. 2018 Oct 25;56(7):4601–19. doi: 10.1007/s12035-018-1383-3 (PMC6657433; doi:10.1007/s12035-018-1383-3)

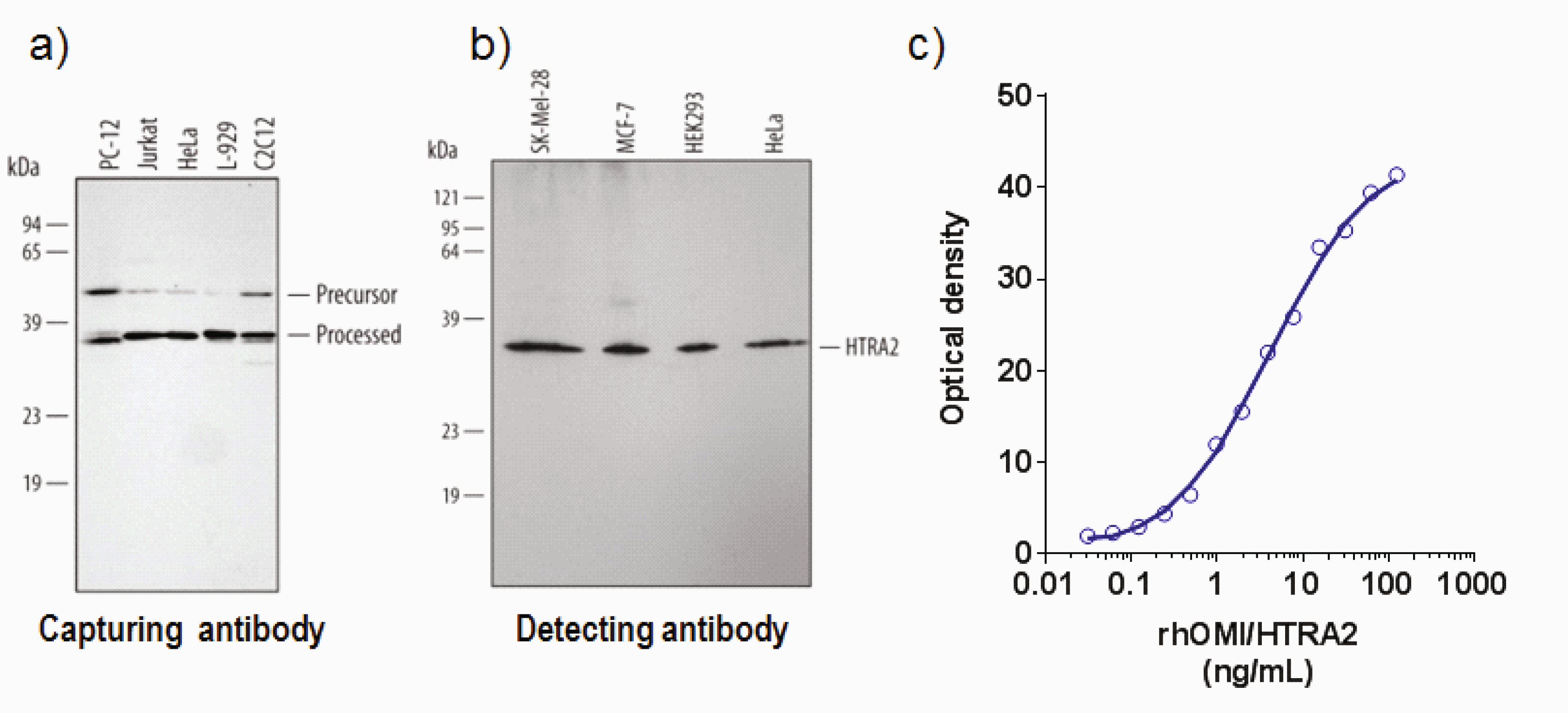

Supplement: Supplementary file 1 — Assay controls for the in-house developed sandwich ELISA specific for the activated form of OMI/HTRA2. A sandwich ELISA assay was setup and used to quantify the protein level of activated OMI/HTRA2 in the brain extracts from three different brain regions of patients and controls. OMI/HTRA2 protein was adsorbed to the wells of an ELISA plate, pre-coated by the capturing antibody, rabbit polyclonal anti-OMI/HTRA2 antibody (AF1458; R&D systems). This antibody detects both the precursor (unprocessed, 49-50 kDa) and the processed (activated, ~36 kDa) bands of OMI/HTRA2 protein under reducing conditions of western blotting (a). Then the mouse monoclonal anti-OMI/HTRA2 antibody (MAB1458, R&D System) was used, which only detects the activated form of the OMI/HTRA2 protein (b). A comparison of blot a, and b suggests that the detecting antibody requires epitope exposure available in the processed form of OMI/HTRA2 (activated form). The graph in (c) illustrate a representative standard curve of a serial dilution of recombinant human OMI/HTRA2 protein (1458-HT; R&D systems) used in the in-house ELISA to quantify the activated form of OMI/HTRA2 ELISA assay. In a typical assay setup, the concentration of rhOMI/HTRA2 standard protein ranged between 125 ng/mL to 0.031 ng/mL, and the optical density of OMI/HTRA2 protein in the brain extracts were in the middle of the standard curve. Additional details are described in the Material and Method section. The western blots were obtained from R&D System web-site with their permission. (PNG 360 kb) [file 12035_2018_1383_Fig11_ESM.png]

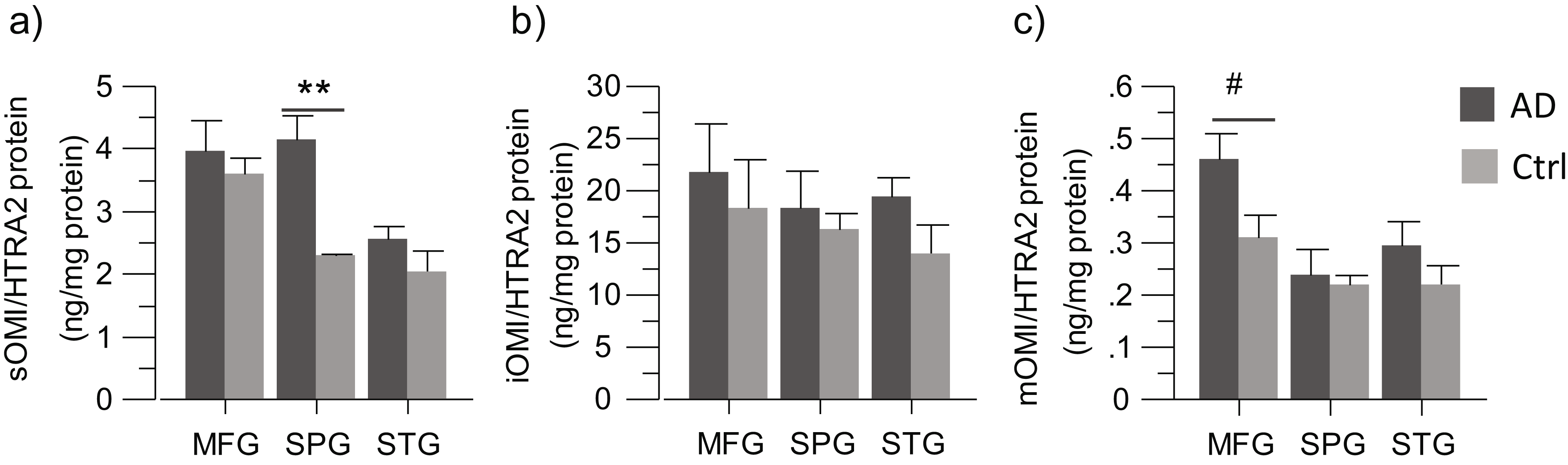

Supplement: Supplementary file 3 — Levels of activated OMI/HTRA2 in different brain extracts. Three different brain extracts were consecutively prepared using different homogenization buffers to extract soluble (s), ionic (i) and membrane bound (m) proteins. The extracts were prepared from three brain regions, namely from MFG (medial frontal gyrus), STG (superior temporalis gyrus) and SPG (superior parietal gyrus) regions. Post-mortem brain tissues were from six AD and six non-demented controls. Activated OMI/HTRA2 protein levels were quantified and defined as soluble (s), ionic (i), and membrane-bound (m) OMI/HTRA2 protein extracts), by an in house sandwich ELISA as described in the “Material and Method” section (graphs a, b, c, respectively). All values are normalized to the total protein in the extracts and are expressed as ng of OMI/HTRA2 protein/mg of total protein. Noteworthy, the absolute majority of activated OMI/HTRA2 protein was found in ionic brain extract. **p <0.01;#p <0.06. (PNG 126 kb) [file 12035_2018_1383_Fig12_ESM.png]
