# Supplementary material for: Increased Active OMI/HTRA2 Serine Protease Displays a Positive Correlation with Cholinergic Alterations in the Alzheimer’s Disease Brain
Source: Mol Neurobiol. 2018 Oct 25;56(7):4601–19. doi: 10.1007/s12035-018-1383-3 (PMC6657433; doi:10.1007/s12035-018-1383-3)
Supplement: Supplementary file 5 — (DOCX 17 kb) [file 12035_2018_1383_MOESM3_ESM.docx]

**Table S1. Multiple regression analysis of the gene expression from the GEO dataset GSE1297.**

| **Global cognition ( as MMSE)**  versus  **9 independents** | **Std. Coefficient** | ***p* <** |
| --- | --- | --- |
| 203089_s_at OMI/HTRA2 | 0.84 | 0.0004 |
| 221197_s_at CHAT | 0.60 | 0.001 |
| 210123_s_at CHRNA7 | 0.35 | 0.027 |
| 205377_s_at ACHE | -0.42 | 0.014 |
| 206401_s_at MAPT | 0.50 | 0.002 |
| 203929_s_at MAPT | -0.41 | 0.0091 |
| 214953_s_at APP | -0.41 | 0.031 |
| 211277_x_at APP | -0.48 | 0.010 |
| 200602_at APP | 0.48 | 0.013 |

Individual r-values are represented as standard coefficient.

The overall standard coefficient and *p* value of the analysis was 0.84 and <0.0006, respectively. MMSE= Mini Mental State Examination, a measure of global cognition.

The GEO dataset GSE1297 contains gene expression data from hippocampus of 9 controls (MMSE 28±1, age 85±3 years) and 22 AD (MMSE 17±2, age 86±2 years) at various pathological stages (Incipient n=7, MMSE 24±1; Moderate n=8, MMSE 17±1; and Severe n=7, MMSE 6±1)**.**

**Table S2. Multiple regression analysis of the gene expression from the GEO dataset GSE1297.**

| **203089_s_at OMI/HTRA2**  versus  **4 independents** | **Std. Coefficient** | ***P*<** |
| --- | --- | --- |
| 221197_s_at CHAT | -0.37 | 0.0030 |
| 205377_s_at ACHE | 0.65 | 0.0001 |
| 200602_at APP | -0.46 | 0.0004 |

Individual r-values are represented as standard coefficient.

The overall standard coefficient and *p* value of the analysis was 0.82 and <0.0001, respectively.
